# Raman and X-ray diffraction study of pressure-induced phase transition in synthetic Mg_2_TiO_4_

**DOI:** 10.1038/s41598-020-63202-5

**Published:** 2020-04-14

**Authors:** Ching-Pao Wang, Sean R. Shieh, Anthony C. Withers, Xi Liu, Dongzhou Zhang, Sergey N. Tkachev, Abd-Erraouf Djirar, Tianqi Xie, Justin D. Rumney

**Affiliations:** 1 Department of Earth Sciences, University of Western Ontario, London, Ontario N6A 5B7 Canada; 2 Department of Physics and Astronomy, University of Western Ontario, London, Ontario N6A 5B7 Canada; 3 Bayerisches Geoinstitut, Universität Bayreuth, Bayreuth, Germany; 4 Key Laboratory of Orogenic Belts and Crustal Evolution, MOE, Peking University, Beijing, 100871 China; 5 School of Earth and Space Sciences, Peking University, Beijing, 100871 China; 6 School of Ocean and Earth Science and Technology, Hawai’i Institute of Geophysics and Planetology, University of Hawaii at Manoa, Honolulu, HI 96822 United States; 7 Center for Advanced Radiation Sources, University of Chicago, Chicago, Illinois 60637 United States

**Keywords:** Transformation optics, X-rays

## Abstract

Synthetic Mg_2_TiO_4_ qandilite was investigated to 50 and 40.4 GPa at room temperature using Raman spectroscopy and X-ray diffraction, respectively. The Raman measurements showed that cubic Mg_2_TiO_4_ spinel transforms to a high pressure tetragonal (I4_1_/amd, No.141) phase at 14.7 GPa. Owing to sluggish kinetics at room temperature, the spinel phase coexists with the tetragonal phase between 14.7 and 24.3 GPa. In the X-ray diffraction experiment, transformation of the cubic Mg_2_TiO_4_ to the tetragonal structure was complete by 29.2 GPa, ~5 GPa higher than the transition pressure obtained by Raman measurements, owing to slow kinetics. The obtained isothermal bulk modulus of Mg_2_TiO_4_ spinel is *K*_T0_ = 148(3) GPa when *K*_T0_’ = 6.6, or *K*_T0_ = 166(1) GPa when *K*_T0_’ is fixed at 4. The isothermal bulk modulus of the high-pressure tetragonal phase is calculated to be 209(2) GPa and V_0_ = 270(2) Å^3^ when *K*_T0_’ is fixed at 4, and the volume reduction on change from cubic to tetragonal phase is about 9%. The calculated thermal Grüneisen parameters (γ_*th*_) of cubic and tetragonal Mg_2_TiO_4_ phases are 1.01 and 0.63. Based on the radii ratio of spinel cations, a simple model is proposed to predict post-spinel structures.

## Introduction

Mg_2_TiO_4_ (qandilite) is an oxospinel with excellent dielectric properties that are widely used in satellite communications, mobile phones and wireless communication systems^1,2^. In addition, Mg_2_TiO_4_ spinel can form a high-temperature superconducting epitaxial thin film^3,4^, and is a good candidate for thin film phosphor in optoelectronic applications due to its red emission at high temperature^5^. Natural Mg_2_TiO_4_ was discovered in the Kangerdlugssuaq region of East Greenland^6^ and named qandilite after the Qandil Group of metamorphic rocks at Qala-Dizeh region of Iraq^7^. At ambient pressure, Mg_2_TiO_4_ exhibits as a tetragonal structure below 660 °C but as a cubic structure above 660 °C^8,9^. The cubic phase breaks down to MgTiO_3_ (geikielite) and MgO (periclase) with increasing pressure^10^. Synthetic Mg_2_TiO_4_ qandilite has inverse spinel structure ^T^(Mg^2+^)°(Mg^2+^, Ti^4+^)O_4_^9,11,12^, which means that Mg^2+^ cations occupy both tetrahedral (T) and octahedral (O) sites, while Ti^4+^ cations are present only in octahedral sites. The isothermal bulk modulus of cubic qandilite was reported to be 169 GPa, based on empirical calculations^13^, and 175 GPa in a diamond anvil cell study^14^, whereas the adiabatic bulk modulus of qandilite was determined to be 152 GPa by ultrasonic measurements^15^. However, no phase transformation was found in previous studies, even though three major post-spinel structures, namely CaTi_2_O_4_ (CT; space group *Cmcm*), CaMn_2_O_4_ (CM; space group *Pbcm*), and CaFe_2_O_4_ (CF; space group *Pnma*) have been proposed to be stable under high pressure environments^16^. Unlike CM and CF structures, most CT phases have not been discovered at room temperature but instead under higher temperatures^17–19^. In addition to the orthorhombic structures, a tetragonal structure (space group I4_1_/amd) was reported for post-spinel phases at high pressure conditions as an intermediate phase^20–25^. The post-spinel structures attract considerable attention because they are isostructural with ringwoodite, which is the most abundant phase, comprising approximately 50–60% by volume, in the Earth’s transition zone (400–600 km in depth)^26^. Furthermore, knowledge of the post-spinel phase may have important implications for structure and dynamics of the interior of the exoplanets. Because of the geological and material importance of Mg_2_TiO_4_ phases, high pressure measurements of phase stability, structure determination, and thermodynamic properties are needed in the Mg_2_TiO_4_ system. In this study, *in situ* high- pressure Raman measurements and equations of states of both spinel and post-spinel structures of synthetic Mg_2_TiO_4_ qandilite were investigated at room temperature. The post-spinel phase was identified and a model for prediction of post-spinel structure is also reported.

## Result and Discussion

**Figure 1:**
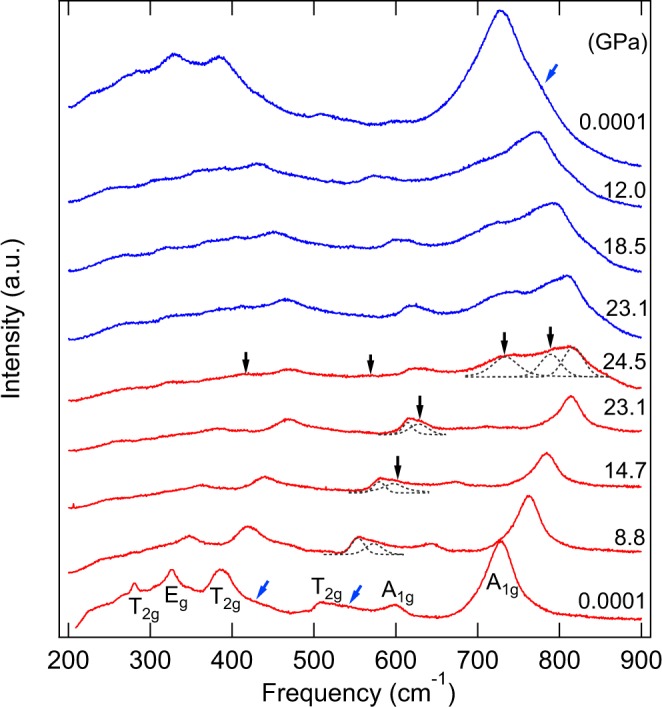
Raman spectra of Mg_2_TiO_4_ at pressure to 24.5 GPa and room temperature. A new Raman peak appeared at 14.7 GPa and four additional modes of the high-pressure phase appeared at 24.5 GPa. Red: compression, blue: decompression. Blue and black arrows indicate the Raman modes of spinel and tetragonal phases, respectively.

**Figure 2:**
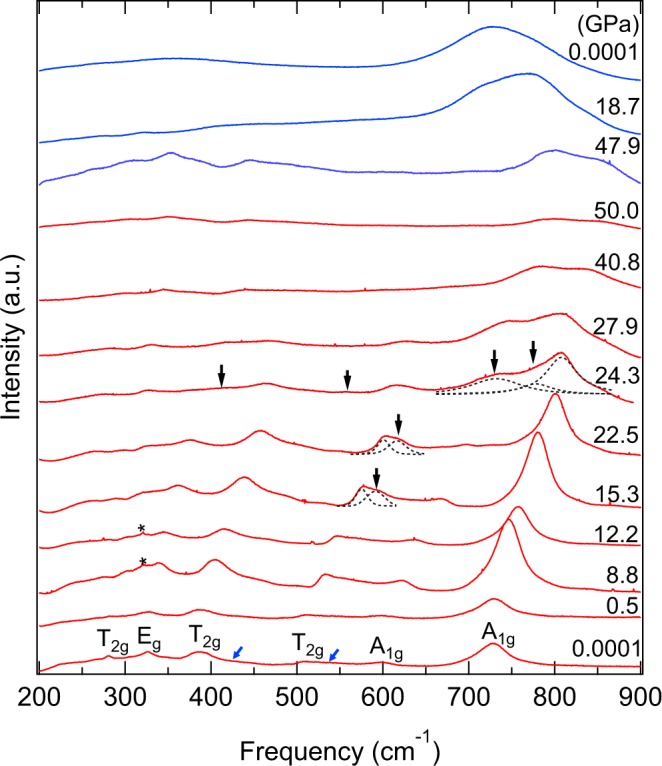
Raman spectra of Mg_2_TiO_4_ at pressure to 50.0 GPa and room temperature. A new Raman mode appeared at 15.3 GPa and four new peaks appeared at 24.3 GPa. Red: compression, blue: decompression, asterisk symbols (*) only observed at 8.8 and 12.2 GPa are MgTiO_3_. Blue arrows are the shoulders of Mg_2_TiO_4_ Raman modes. Blue and black arrows indicate the Raman modes of spinel and tetragonal phases, respectively.

Two Raman measurements were conducted at pressure to 24.5 GPa and 50 GPa, respectively. Group theory predicts that for normal spinels at the Γ point of the Brillouin zone^27^:

$$\Gamma ={{\rm{A}}}_{1{\rm{g}}}({\rm{R}})+{{\rm{E}}}_{{\rm{g}}}({\rm{R}})+{{\rm{T}}}_{1{\rm{g}}}+3{{\rm{T}}}_{2{\rm{g}}}({\rm{R}})+2{{\rm{A}}}_{u}+{{\rm{E}}}_{u}+4{{\rm{T}}}_{1u}+{{\rm{T}}}_{1u}+2{{\rm{T}}}_{2u}$$

where R denotes Raman-active modes and u denotes infrared-active modes. Therefore, five Raman bands are predicted for the normal spinels. However, cation disorder in the inverse spinel is expected to cause splitting of the A_1g_ mode^28–31^. In this study, two A_1g_, one E_g_, and three T_2g_ were observed for Mg_2_TiO_4_ spinel, and, in addition, two weak peaks at the shoulders of T_2g_ (432 and 546 cm^−1^) were present (blue arrows in Fig. 1). The higher frequency peaks of A_1g_ modes are assigned to stretching of the MgO_4_ tetrahedron and the split A_1g_ mode is found at about 596 cm^−1^ in our measurements. The E_g_ mode corresponds to a symmetric bending vibration of the oxygens within tetragonal units. The T_2g_ near 506 cm^−1^ is caused by an asymmetric bending of O-Mg-O bonds and the other T_2g_ near 281 cm^−1^ is assigned to the translation between TiO_6_ octahedron and Mg cation. The T_2g_ near 385 cm^−1^ arises from the opposing translations between cations and oxygens along one direction of the lattice. The two different shoulders of T_2g_ are likely related to cation substitutions. When Mg_2_TiO_4_ spinel was compressed to 14.7 GPa, a new peak appeared close to 595 cm^−1^. Upon further compression to 24.5 GPa, four additional new peaks were observed, strongly suggesting a new phase. To test whether the new high-pressure phase can be quenchable, the sample was gradually decompressed to ambient pressure, after which a shoulder of high frequency A_1g_ from the high pressure phase was retained, suggesting that the new phase did not fully back-transform and some amount of the new phase coexisted with the spinel phase (Fig. 1). The second Raman measurements were conducted to 50 GPa and the results are shown in Fig. 2. Phase transition again occurred near 15 GPa, marked by the appearance of a single new peak. The full spectrum of the new phase was evident at 24.3 GPa, in agreement with the first run, and the new phase persisted to 50 GPa. However, upon decompression the high-pressure phase was retained to about 18.7 GPa and most of the features were diminished with further decompression, except for the broad A_1g_ peak near 700–800 cm^−1^. Minor MgTiO_3_ impurity was also observed during the second Raman measurement at 8.8 and 12.2 GPa, giving rise to the weak feature marked by an asterisk in the second set of Raman measurements. A previous Raman study of MgTiO_3_ showed no phase change at pressure to 27 GPa^32^, suggesting that even if there is minor MgTiO_3_ impurity, it should not have any contribution to the transition pressure.

**Figure 3:**
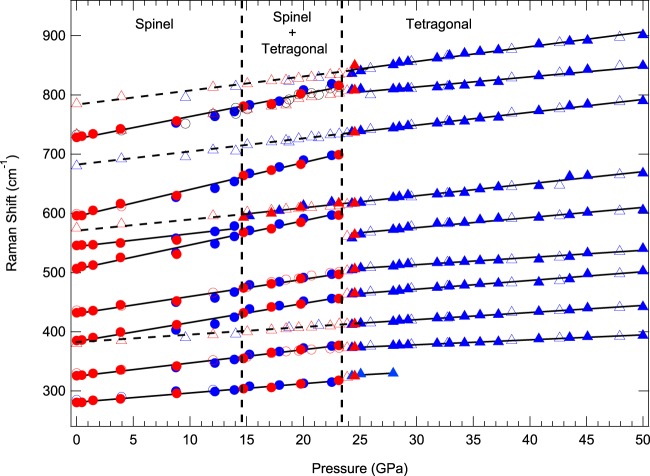
Raman frequency shifts as a function of pressure for Mg_2_TiO_4_ to 50.0 GPa. A new peak appears close to 595 cm^−1^ at 14.7 GPa and four new Raman modes appear at 24.3 GPa. Solid symbols are compression data whereas the open symbols represent decompression. Circles denote spinel phase, and triangles the tetragonal phase. Red symbols denote the first run to 24.5 GPa and blue symbols represent the second run to 50 GPa. The error bars of all data points are smaller than the symbols.

To better understand the phase transition boundary, the frequency shifts as a function of pressure are plotted in Fig. 3. A single new peak observed at slightly higher frequency near 595 cm^−1^ at about 14.7 GPa and is assigned to the new phase. A discontinuity was clearly observed near 24.3 GPa, as evidenced by four additional modes near 413, 558, 737, and 836 cm^−1^. Our Raman data suggest that the new phase only partially transformed at about 14.7 GPa, and that the transformation is very sluggish. A two-phase mixture therefore persists between 14.7 and 24.3 GPa. The decompression results showed that the high-pressure phase was partly quenchable, and both spinel and high-pressure phases coexisted to ambient pressure. Four high-pressure modes and six cubic bands were found in the quenched Raman spectra based on two sets of Raman measurements, but the peak at about 500 cm^−1^, which coincides with the position of a peak in compression data, appeared only below 4 GPa. Since this is a first-order phase transition for Mg_2_TiO_4_, we were then able to obtain the slopes of pressure dependence dν_i_/dP values based on two different datasets of curve fits.

The high-pressure phase of Mg_2_TiO_4_ could have a tetragonal structure, based on the splitting of MgO_4_ modes. Previous studies on ZnGa_2_O_4_^20^ and MgCr_2_O_4_^22^ also suggested a cubic to tetragonal transition at high pressure and room temperature. Our X-ray diffraction analyses (see below) suggest that the high-pressure phase is a tetragonal spinel (I4_1_/amd, No.141). The new peak at 413 cm^−1^ splits from E_g_ at 24.3 GPa due to the different bending vibration of oxygen at tetrahedral units, and the other new peak at 558 cm^−1^ appeared at high pressure in the tetragonal phase because of the change of O-Mg-O bending from original higher-frequency T_2g_. The cubic spinel A_1g_ peak at 737 cm^−1^ split into two peaks at 809 and 836 cm^−1^, which can be attributed to differences in the shortening of bond lengths of the MgO_4_ tetrahedron. As a result, the observed Raman-mode frequencies (*ν*), pressure dependencies (d*ν*_i_/d*P*), and mode Grüneisen parameters (*γ*_i_) for spinel and for the high-pressure phase are listed in Table 1. The pressure dependencies dν_i_/dP of Mg_2_TiO_4_ spinel indicate that two higher-frequency T_2g_ and one A_1g_ are more compressible, and the lowest-frequency T_2g_ are stiffer. The lowest-frequency E_g_ of the high-pressure phase is stiffer than the other Raman modes. Mode Grüneisen parameters (γ_i_) are calculated from the equation *γ*_i_ = $\frac{{K}_{{\rm{T}}}}{{{\rm{\nu }}}_{0}}{\left(\frac{{\rm{d}}{\nu }_{{\rm{i}}}}{{\rm{d}}P}\right)}_{{\rm{T}}}$^33^, where isothermal bulk moduli *K*_T_ of spinel and tetragonal phase are both obtained from this study (see below). The thermal Grüneisen parameter (*γ*_th_)^34,35^ can be calculated as the weighted average of the mode Grüneisen parameters (γ_i_), which are listed in Table 1. The thermal Grüneisen parameter is given by ${\gamma }_{{\rm{th}}}=\frac{{\Sigma }_{i}{C{\rm{v}}}_{i}{{\Upsilon }}_{i}}{{\Sigma }_{i}{C{\rm{v}}}_{i}}$, and the harmonic heat capacity *C*v_*i*_ was estimated from the Einstein function:

$${C{\rm{v}}}_{i}=\kappa {\left(\frac{{h\nu }_{i}}{\kappa T}\right)}^{2}{\rm{\exp }}\left(\frac{{h\nu }_{i}}{\kappa T}\right)/{[{\rm{\exp }}\left(\frac{{h\nu }_{i}}{\kappa T}\right)-1]}^{2}$$

where temperature *T* is 300 kelvins, *h* is the Plank constant, and $\kappa$ is the Boltzmann constant. Our results show that the thermal Grüneisen parameter is 1.01 for the spinel phase and 0.63 for the tetragonal phase.

**Table 1:** Observed Raman-mode frequencies (ν), pressure dependences (dν/dP), and calculated mode Grüneisen parameters (γ_i_) for Mg_2_TiO_4_ at pressure to 50 GPa.

Spinel Phase				High-Pressure Phase		
Mode	ν (cm^−1^)	dν/dP (cm^−1^/GPa)	γ_i_	ν (cm^−1^)	dν/dP (cm^−1^/GPa)	γ_i_
T_2g_	281	2.15	1.13	325	1.08	0.70
E_g_	326	2.24	1.02	373	0.83	0.49
				413	1.20	0.65
T_2g_	385	3.22	1.24	464	1.47	0.72
	432	2.90	0.99	504	1.22	0.53
T_2g_	506	4.08	1.19	558	1.71	0.68
	546	2.17	0.59	617	2.04	0.75
A_1g_	596	4.54	1.13	737	2.18	0.67
A_1g_	728	3.77	0.77	809	1.71	0.47
				836	2.50	0.67

The high-pressure tetragonal phase was collected from 24.3 GPa to 50 GPa.

**Figure 4:**
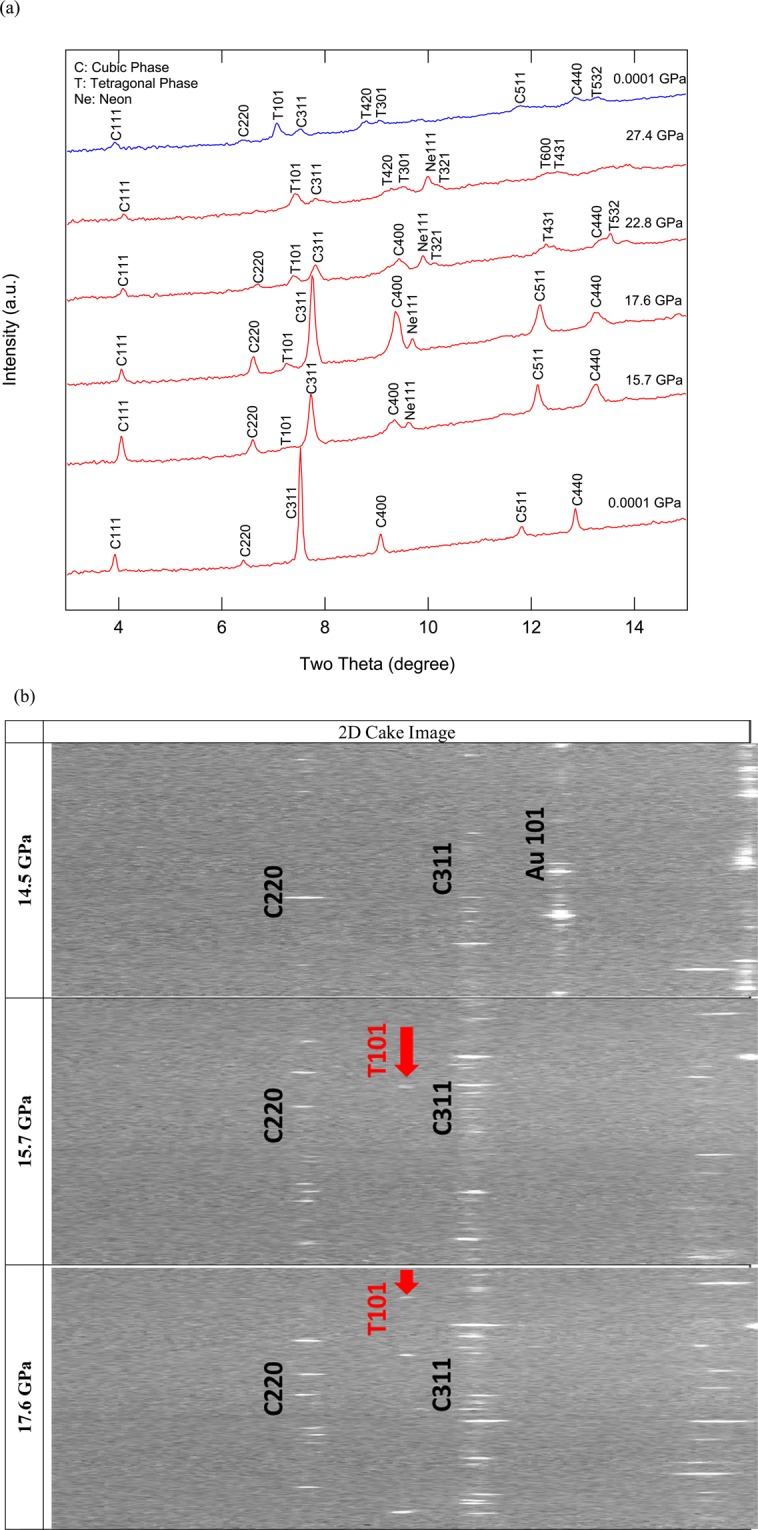
(**a**) Representative X-ray diffraction patterns of Mg_2_TiO_4_ collected in Run 1. A new peak 101 belongs to the high-pressure phase that coexisted with spinel from 15.7 to 27.4 GPa, and is also present in the decompressed pattern. Red; compression patterns, blue: decompression pattern. (**b**) 2D cake images show the new peak T101 observed above 15.7 GPa.

**Figure 5:**
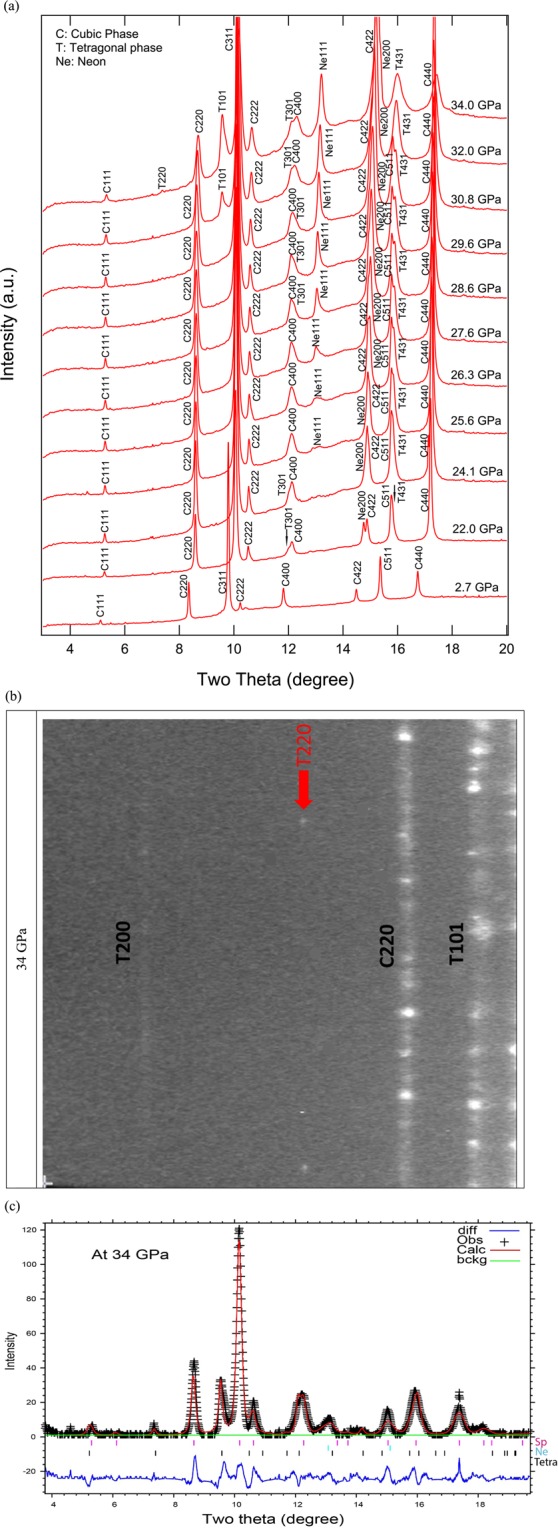
(**a**) Representative X-ray diffraction patterns of Mg_2_TiO_4_ collected in Run 2. The high-pressure tetragonal phase coexisted with low pressure phase at 22–34 GPa, but the T101 of high-pressure phase appeared from 32 GPa and T220 appeared at 34 GPa. (b) 2D cake image showing the new T220 pattern at 34 GPa. (**c**) Le Bail refinement of X-ray diffraction pattern at 34 GPa. The ticks represent three calculated structures: Tetra – high-pressure tetrahedral phase, Sp- ambient pressure cubic phase and Ne – pressure medium.

**Figure 6:**
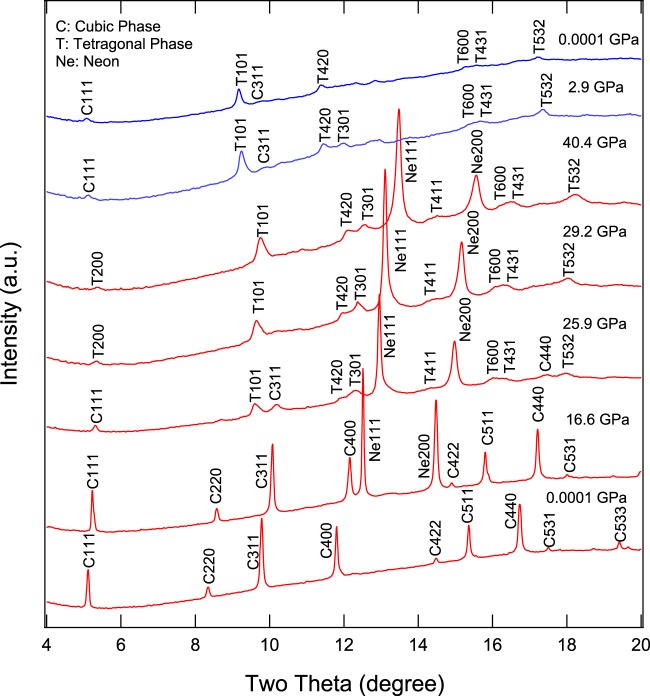
Representative X-ray diffraction patterns of Mg_2_TiO_4_ collected in Run 3. Cubic Mg_2_TiO_4_ spinel fully transforms to tetragonal structure at 29.2 GPa. Upon decompression, both low- and high-pressure phases were present at ambient pressure. The room-pressure pattern was collected after 24 hours of decompression. (Red: compression patterns; blue: decompression patterns).

No MgTiO_3_ contaminant was observed during any of the X-ray diffraction measurements and the ambient-pressure unit-cell lattice parameters of synthetic Mg_2_TiO_4_ spinel collected at both 13-BM-D and 13-BM-C were confirmed to be the pure phase. The ambient-pressure unit-cell parameters of the synthetic Mg_2_TiO_4_ spinel are a_0_ = 8.4464(2) Å and V_0_ = 602.59(5) Å^3^, both of which are comparable with previous studies^9,11,14,36^. *In-situ* high-pressure X-ray diffraction patterns of Mg_2_TiO_4_ to 27.4 GPa collected at beamline 13-BM-D (Run 1) are shown in Fig. 4a. Our two-dimensional images displayed a new feature at 15.7 GPa which could be the high-pressure tetragonal phase T101 (Fig. 4b). At pressure above 22.8 GPa, additional new peaks were observed, allowing us to determine that the structure of the high-pressure phase is tetragonal I4_1_/amd (No. 141). Note that the spinel structure was found to coexist with this high-pressure tetragonal phase, but its diffraction peaks became weaker above 27.4 GPa. As a consequence, no further data at higher pressures were collected from Run 1. Upon decompression, spinel and the tetragonal phases were found to coexist at all pressures to ambient conditions. For Run 2, the pressure was increased directly from 2.7 to 22 GPa and then gradually compressed to 34 GPa within 3 hours (Fig. 5a). Again, the high-pressure tetragonal phase coexisted with Mg_2_TiO_4_ spinel from 22 to 34 GPa and the refined structure at 34 GPa is shown in Fig. 5c. However, the tetragonal phase T101 appeared at 32 GPa, which was about 18 GPa higher than Run 1, and another new peak T220 appeared at 34 GPa (Fig. 5b). For Run 3, the pressure was gently increased from 1 bar to 40.4 GPa over 9 hours (Fig. 6). At 29.2 GPa the cubic spinel had fully transformed to the tetragonal phase. After decompression, both spinel and high-pressure tetragonal phases were retained and persisted for at least 24 h after decompression to ambient conditions. The tetragonal phase T220 is critical for the structure determination but it is actually very weak. We checked all our 2D images and found the T220 only observable at 34 GPa in Run 2. In addition, for the tetragonal phase T532, it only can be assigned to the tetragonal phase by doubling the c-axis to 5.446 Å at 25.9 GPa in Run 3. In summary, our X-ray diffraction results suggest that the tetragonal phase transformation started at 15.7 GPa and completed at 29.2 GPa. In addition, in all three runs, the coexistences of spinel and tetragonal phases over variable ranges of pressure are likely to be a result of sluggish kinetics. Our X-ray diffraction data showed both spinel and tetragonal phases were present between 15.7 and 29.2 GPa, but in the Raman measurements the mixture of phases is limited to a smaller pressure range. We postulate that this is owing to longer collection time in Raman measurements than in the X-ray diffraction measurements and perhaps because local atomic bonding distortions and variations detected by Raman scattering are more sensitive than the bulk structural changes measured using the X-ray diffraction method.

**Figure 7:**
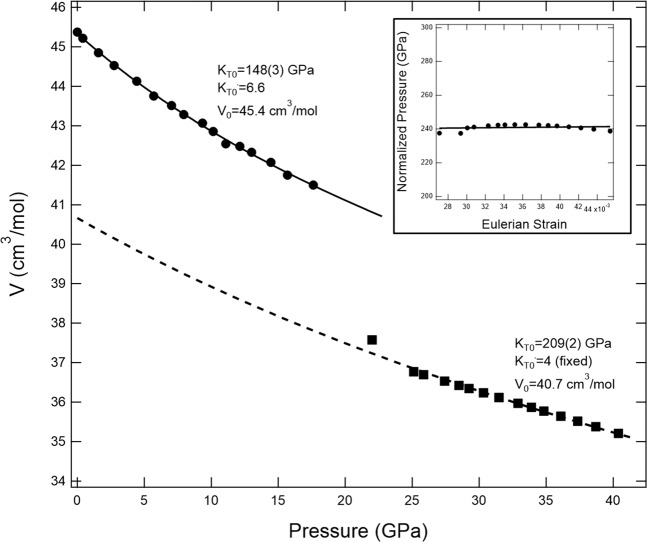
The molar volume as a function of pressure for Mg_2_TiO_4_ to 40 GPa. Solid circles represent volumes of cubic phase and solid squares are from high-pressure tetragonal phase. The solid and dashed curves are best-fit third-order Birch-Murnaghan equations of state. The error bars of all data points are smaller than the symbols. The Eulerian strain-normalized pressure plot shows that *K*_T0_’ of the high-pressure phase is close to 4.

The unit-cell lattice parameters and volume data of Mg_2_TiO_4_ at pressure to 40.4 GPa are summarized in Table 3. The Mg_2_TiO_4_ volume data with respect to pressures were fitted with a third-order Birch-Murnaghan equation of state, with all volume data expressed as molar volumes (Fig. 7). The obtained isothermal bulk modulus of Mg_2_TiO_4_ spinel is *K*_T0_ = 148(3) GPa when *K*_T0_’ = 6.6, and *K*_T0_ = 166(1) GPa when *K*_T0_’ is fixed at 4, in agreement with previous studies^13,14^. The volume change between spinel and tetragonal phase is about 9%. The unit-cell lattice parameters of tetragonal phase obtained from this study are also listed in Table 2. The bulk modulus *K*_T0_ of the tetragonal phase is obtained as 209(2) GPa and V_0_ = 270(2) Å^3^ when *K*_T0_’ is fixed at 4, based on the trend of Eulerian strain-normalized pressure plot^37^.

**Table 2:** Unit-cell lattice parameters of Mg_2_TiO_4_ for spinel and tetragonal phase obtained at high pressures.

Spinel Structure			Tetragonal Structure			
P (GPa)	a(Å)	V(Å^3^)	P (GPa)	a(Å)	c(Å)	V(Å^3^)
0.0001	8.4464(3)	602.59(5)	25.1(1)	9.4686(5)	2.7233(3)	244.16(4)
0.4(1)	8.4366(2)	600.49(5)	25.9(1)	9.4598(5)	2.7228(3)	243.65(4)
1.6(1)	8.4137(2)	595.62(5)	27.4(1)	9.4410(5)	2.7216(3)	242.58(4)
2.7(1)	8.3935(2)	591.33(5)	28.5(1)	9.4282(5)	2.7208(3)	241.85(4)
4.4(1)	8.3684(2)	586.04(5)	29.2(1)	9.4192(5)	2.7202(3)	241.34(4)
5.7(1)	8.3447(2)	581.08(5)	30.3(1)	9.4062(5)	2.7194(3)	240.60(4)
7.0(1)	8.3293(2)	577.87(5)	31.5(1)	9.3923(5)	2.7185(3)	239.81(4)
7.9(1)	8.3146(1)	574.81(5)	32.9(1)	9.3753(5)	2.7174(3)	238.85(4)
9.3(1)	8.3024(1)	571.94(5)	33.9(1)	9.3632(5)	2.7166(3)	238.17(4)
10.1(1)	8.2871(1)	569.12(5)	34.8(1)	9.3521(5)	2.7159(3)	237.54(4)
11.1(1)	8.2670(2)	564.99(5)	36.1(1)	9.3369(5)	2.7150(3)	236.68(4)
12.1(1)	8.2629(2)	564.15(5)	37.4(1)	9.3217(5)	2.7140(3)	235.83(4)
13.0(1)	8.2530(2)	562.13(5)	38.7(1)	9.3055(5)	2.7130(3)	234.92(4)
14.4(1)	8.2364(2)	558.74(5)	40.4(1)	9.2851(5)	2.7117(3)	233.79(4)
15.7(1)	8.2154(2)	554.48(5)				
17.6(1)	8.1987(2)	551.10(5)				

Numbers within parenthesis showed uncertainty of the last digit

**Table 3:** Comparison of the radii of cations, volumes, unit-cell parameters, and bulk moduli of different spinels.

Compound	r_a_ (Å)	r_b_ (Å)	V(Å^3^)	a (Å)	K_0_ (GPa)	K_0_’	HP Phase	Reference
FeCr_2_O_4_	0.78	0.615	588	8.378	209	4.0	I4_1_/amd	^21^
Fe_3_O_4_	0.78	0.645	591.4	8.394	182	3.6	*Pbcm*	^54^
Fe_2_TiO_4_	0.605	0.645	624.3	8.530	250.8	4.0	*Cmcm*	^38^
MgAl_2_O_4_	0.72	0.535	507.8	7.978	212	6.3	*Pnma*	^55^
MgCr_2_O_4_	0.72	0.615	578.7	8.333	189	7.2	I4_1_/amd	^22^
Mg_2_TiO_4_	0.605	0.72	602.6	8.446	166	4	I4_1_/amd	This Study
MgFe_2_O_4_	0.72	0.645	589.9	8.387	195	4	*Pbcm*	^40^
MnFe_2_O_4_	0.83	0.645	617.5	8.5157	169.7	2.87	*Pbcm*	^42^
NiMn_2_O_4_	0.69	0.645	590.6	8.390	206	4	I4_1_/amd	^45^
ZnAl_2_O_4_	0.74	0.535	529.7	8.091	201.7	7.62	*Pnma*	^56^
ZnGa_2_O_4_	0.74	0.62	580.1	8.340	233	8.3	I4_1_/amd	^57^
Zn_2_TiO_4_	0.605	0.74	608.2	8.472	154.6	4	I4_1_/amd	^48^
Co_2_TiO_4_	0.605	0.75	604.38	8.454	167.2	4	*Pbcm*	^58^

**Figure 8:**
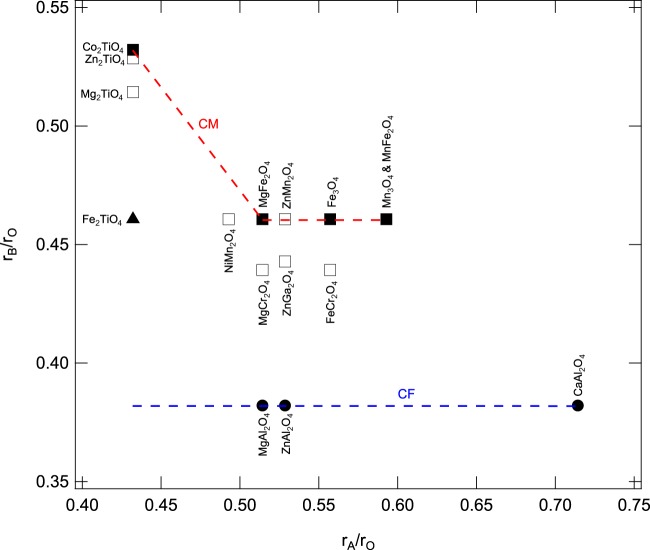
The radius ratio r_A_/r_O_ versus r_B_/r_O_. for different spinels. Four different post-spinel structures, CT (solid triangle), CF (solid circle), tetragonal (open square), and CM (solid square), are shown. There are two curves which show the trends of CM (red) and CF (blue) post-spinel phases, respectively. The trend of CF at lower r_A_/r_O_ is calculated from^47^. Different post-spinel phases lie in the small region of r_A_/r_O_ < 0.45 and r_B_/r_O_ > 0.5. The regions of CM and tetragonal are quite close, but the trends between CM and CF are distinct.

To evaluate the post-spinel structure for the orthotitanates, we compare several spinel phases and their post-spinel structures such as CT^38^, CM^39–42^, CF^20,41,43,44^, and intermediate phases^20–23,45,46^ (Table 3). The CF phase of AB_2_O_4_ post-spinel was limited by the radius ratio r_B_/r_A_. In general, CF phase can be found in the range of 0.53 to 0.89, but not in the case of r_B_/r_A_ < 0.53^47^. The radius ratio r_B_/r_A_ of Mg_2_TiO_4_ is around 1.19 which is larger than the range of CF phase but very close to Zn_2_TiO_4_ with radius ratio r_B_/r_A_ of 1.22. The post-spinel structure of Zn_2_TiO_4_ was suggested to be CT phase^48^ with an intermediate tetragonal phase^23^. The radii of cations^49^ in tetrahedral and octahedral sites could be a determining factor in the structure of AB_2_O_4_ spinel. Normal spinel has the formula ${{\rm{A}}}^{{\rm{T}}}{{\rm{B}}}_{2}^{{\rm{O}}}{{\rm{O}}}_{4}$, where A cations sit in tetrahedral sites and B cations sit in octahedral ones. If the radius of A cations is too big, they are unlikely to remain in tetrahedral sites. The formula of inverse spinel can be expressed as ${{\rm{B}}}^{{\rm{T}}}{({\rm{A}},{\rm{B}})}^{{\rm{O}}}{{\rm{O}}}_{4}$, where A cations do not sit in tetrahedral sites anymore and the size limit for B cations is smaller as they have to fit into tetrahedral sites. Nevertheless, whether in normal or inverse spinels, A cations are usually larger than B cations under ambient conditions. Figure 8 shows the radius ratio r_A_/r_O_ with respect to r_B_/r_O_ for several different spinels at ambient conditions, and two distinct trends of CM and CF are observed. Intermediate tetragonal and CT phases lie mostly between CM and CF curves. The A cations (Ti^4+^) of Mg_2_TiO_4_, Zn_2_TiO_4_, and Co_2_TiO_4_ inverse spinels are the same and the radii of B cations (Mg^2+^, Zn^2+^, and Co^2+^) are very similar. However, they have different post-spinel structures within the area r_A_/r_O_ < 0.45 and r_B_/r_O_ > 0.5. The possible explanation is that when r_A_ is smaller than r_B_, not only B cations can sit in both tetrahedron and octahedron but also A cations. With the exception of the CF phase, the trends of all post spinels are located within the area where radius ratio r_B_/r_A_ is smaller than 1.

## Conclusion

Two sets of Raman measurements were performed at pressure to 24.5 GPa and 50 GPa in this study. Our Raman results demonstrated that a phase transformation occurred at 14.7 GPa, and a two-phase mixture persisted at pressure up to 24.3 GPa. The cubic Mg_2_TiO_4_ spinel fully transformed to a tetragonal phase above 24.3 GPa and the new phase persisted to 50 GPa. On decompression, the high-pressure phase was observable from 50 to about 18.7 GPa, and most Raman features were diminished with further decompression except the broad A_1g_ peak near 700–800 cm^−1^. The high-pressure phase was partly quenchable below 18.7 GPa, and both spinel and high-pressure phases coexisted to ambient pressure. Our X-ray diffraction data suggest that the tetragonal phase transformation started at 15.7 GPa and completed at 29.2 GPa. The coexisting cubic and tetragonal phases are also shown on our decompression X-ray diffraction patterns at ambient pressure. Comparing our three X-ray diffraction runs, the coexistence of spinel and tetragonal phases extends over three different pressure ranges, and the full phase transformation pressure is ~5 GPa higher than Raman measurements, which is likely the result of slow kinetics. The obtained isothermal bulk modulus of Mg_2_TiO_4_ spinel is *K*_T0_ = 148(3) GPa when *K*_T0_’ = 6.6, or *K*_T0_ = 166(1) GPa when *K*_T0_’ is fixed at 4. The isothermal bulk modulus of high-pressure tetragonal phase is calculated as 209(2) GPa and V_0_ = 270(2) Å^3^ when *K*_T0_’ is fixed at 4, and the volume reduction from cubic to tetragonal phase is about 9%. Grüneisen parameters (γ_*th*_) calculated from the isothermal bulk moduli *K*_T_ of spinel and tetragonal phases obtained from this study are 1.01 and 0.63. A simple model to predict post-spinel structures is proposed based on the radii ratio of spinel cations and our model shows the tetragonal phases located in between the CF and CM trends.

## Experimental Methods

*In situ* high-pressure and room-temperature Raman and X-ray diffraction measurements on Mg_2_TiO_4_ qandilite were performed at pressure to 50 and 40.4 GPa, respectively, using symmetric diamond anvil cells. Mg_2_TiO_4_ qandilite starting material was synthesized at 1673 K for 52 h from a mixture of MgO and TiO_2_. The product was examined by electron probe microanalysis and conventional X-ray diffraction. The results show Mg_2_TiO_4_ with less than 5% of MgTiO_3_^14^. We used a pair of 300-µm diamond culets for both Raman and X-ray diffraction measurements in the high-pressure diamond anvil cell study. Rhenium gaskets were pre-indented to 35–40 µm thickness and a 150-µm hole was drilled to create a sample chamber. Neon was used as a pressure-transmitting medium, together with 1 or 2 ruby spheres for both Raman and X-ray diffraction experiments. A small piece of 10–15 μm gold foil was also loaded in the sample chamber as a pressure marker for the synchrotron X-ray study. Pressure was monitored by the ruby fluorescence method^50^ and/or equation of state of gold^51^.

Raman spectra were collected at the University of Western Ontario using a custom-built system. An argon-ion laser with a wavelength of 514.5 nm was used as an excitation source. The Raman signals were collected by a spectrometer with a 500-mm focal length and equipped with a liquid nitrogen-cooled CCD detector. The spectrometer was calibrated by a neon lamp and a silicon chip. The uncertainty in Raman shift measurements did not exceed 1 cm^−1^. Two different *in situ* Raman measurements were carried out to 24.5 and 50 GPa, respectively. The pressure was measured by the shift of ruby R_1_ emission peak before and after Raman measurements. The collection time of each spectrum was 240 seconds at lower pressure and then increased to 420 seconds at pressure above 24 GPa. The reported spectrum was the average of five spectra at each pressure step. Peakfit software (SPSS Inc., Chicago) was used for Raman peak curve-fitting.

*In situ* high-pressure angular-dispersive X-ray diffraction experiments were carried out at beamline 13-BM-C and 13-BM-D, sectors of GSECARS, Advanced Photon Source. At both beamlines LaB_6_ was used for sample-to-detector distance calibration. The purity of the Mg_2_TiO_4_ spinel phase was confirmed by X-ray diffraction before the high-pressure experiments. One run (Run 1) at pressure to 27.4 GPa was performed at 13-BM-D. The wavelength of the monochromatic X-ray beam at 13-BM-D was 0.3344 Å, and the beam size was focused to 3 × 7 µm. Two runs (Run 2 and 3) at pressures to 34 and 40.4 GPa were conducted at 13-BM-C. The wavelength of the monochromatic X-ray beam at 13-BM-C was 0.434 Å and beam size was focused to 12 ×18 µm. X-ray diffraction patterns were collected by two-dimensional MAR CCD at 13-BM-C and the exposure time of each pattern was 90–120 seconds with sample rotation angle from 80 to 100 degrees. The exposure time of a Perkin-Elmer area detector for each collection at 13-BM-D was 5 seconds without rotation and each diffraction image was the average of 20 patterns to enhance the signal-to-noise ratio. Two-dimensional images were integrated and reduced to one-dimensional patterns using Dioptas software^52^. The powder X-ray diffraction data were processed using the software PeakFit V4.12 (SPSS Inc.) and the unit cell parameters were calculated by the program UnitCell^53^. Experimental powder diffraction results are compared to theoretical values calculated using CRYSTALDIFFRACT software and Le Bail refinement by GSAS (Fig. 5c).
